# New Functionalized Macroparticles for Environmentally Sustainable Biofilm Control in Water Systems

**DOI:** 10.3390/antibiotics10040399

**Published:** 2021-04-07

**Authors:** Ana C. Barros, Ana Pereira, Luis F. Melo, Juliana P. S. Sousa

**Affiliations:** 1 LEPABE—Laboratory for Process Engineering, Environment, Biotechnology and Energy, Faculty of Engineering, University of Porto, 4200-465 Porto, Portugal; aalex@fe.up.pt (A.P.); lmelo@fe.up.pt (L.F.M.); 2 INL, International Iberian Nanotechnology Laboratory, Avenida Mestre José Veiga s/n, 4715-330 Braga, Portugal; juliana.sousa@inl.int

**Keywords:** biocidal particles, functionalization, benzalkonium chloride, *Escherichia coli*, antimicrobial activity

## Abstract

Reverse osmosis (RO) depends on biocidal agents to control the operating costs associated to biofouling, although this implies the discharge of undesired chemicals into the aquatic environment. Therefore, a system providing pre-treated water free of biocides arises as an interesting solution to minimize the discharge of chemicals while enhancing RO filtration performance by inactivating bacteria that could form biofilms on the membrane system. This work proposes a pretreatment approach based on the immobilization of an industrially used antimicrobial agent (benzalkonium chloride—BAC) into millimetric aluminum oxide particles with prior surface activation with DA—dopamine. The antimicrobial efficacy of the functionalized particles was assessed against *Escherichia coli* planktonic cells through culturability and cell membrane integrity analysis. The results showed total inactivation of bacterial cells within five min for the highest particle concentration and 100% of cell membrane damage after 15 min for all concentrations. When reusing the same particles, a higher contact time was needed to reach the total inactivation, possibly due to partial blocking of immobilized biocide by dead bacteria adhering to the particles and to the residual leaching of biocide. The overall results support the use of Al_2_O_3_-DA-BAC particles as antimicrobial agents for sustainable biocidal applications in continuous water treatment systems.

## 1. Introduction

The so-called clean water crisis, which already affects near four billion people [1], is a major humanitarian issue. The effect of unbalanced urbanization, population growth and inadequate use of water is expected to increase between 22% and 34% until 2050 [1]. The requalification or reuse of water from non-potable sources is considered one of the most promising and attractive ways to face such water crisis, with special emphasis on the use of Reverse Osmosis (RO) systems to accomplish proper water “purification”.

Biofouling—the attachment and accumulation of unwanted microorganisms in the form of a biofilm to an extent that interferes negatively with the filtration process [2]—is a major factor contributing to the decrease of the operational performance of RO systems. It is widely consensual that biofouling in RO systems is the main responsible for the decrease in permeate flow rate and quality, increase of pressure drop and of energy demand [3]. Since biofilm build-up is still practically unavoidable, new and sustainable approaches are needed to circumvent this issue [4].

The use of antimicrobial agents, typically disinfectants and/or biocides, is a challenging question across the water treatment sector. On one hand, antimicrobial agents per se are not always able to eradicate biofouling, nor have a similar killing effect on planktonic and sessile (attached) bacteria [5,6]. Additionally, the use of antimicrobial agents dissolved in the water is known to have a negative impact on process economy, health and environment, mostly associated with their discharge along with the rejection stream [7]. On the other hand, it is generally accepted that biocides are important to control microbiological proliferation. They became a “must have” feature to include in RO systems and are used not only in corrective procedures but, also, in routine-based operations [8].

This paradigm opened an opportunity to explore different ways of keeping systems microbiologically controlled while reducing the environmental and public health impacts associated with the use of biocides as a daily-basis procedure. The rise of nanotechnology and the possibility to aggregate new physicochemical properties to the materials in terms of surface area, topography and chemical function [9] is being extensively explored to fulfil this challenge. Several of the approaches that are being investigated include the development of materials with self-antimicrobial properties—e.g., nano-Ag, nano-ZnO, etc. [10,11,12,13,14,15,16,17,18]—and the immobilization of biocidal agents on nanostructured materials due to their high surface-area-to-volume ratio and surface modifiability [19,20,21,22]. These materials can be used as adsorbents of pollutants or as carriers for the biocidal agent. However, serious environmental and health concerns are arising about the fate and toxicity of nanoparticles in water systems [23].

In the present work, a different approach is proposed: (i) the antimicrobial particles are pellets with overall millimetric size, which can be packed in a particle bed system through which the water containing microorganisms flows continuously; (ii) due to their relatively larger dimensions (10^6^ times the size of nanomaterials), these particles can be easily contained in a vessel and not carried away with the outlet water stream. As regards the composition of the particles, metal oxides were chosen, since they have already been successfully applied for environmental monitoring, remediation and pollution prevention [24] and have a low cost. Commercial metal oxides, namely γ-alumina, present excellent mechanical and chemical properties; however, their surface chemical properties do not favor the direct immobilization of biocides. Therefore, it is necessary to previously activate the surfaces to increase their surface reactivity. Herein, an easy and reproducible approach to immobilize biocides onto the surface of metal oxides is proposed. First, the metal oxide particles are functionalized with a precursor to activate their surface reactivity and then the biocide is immobilized onto the surface by putting the metal oxide particles in contact with the biocidal solution.

In this work, alumina particles were functionalized by using a nitrogen precursor (Dopamine (DA)). Subsequently, the biocide benzalkonium chloride (BAC) was immobilized in these particles, and their antimicrobial activity was assessed against *Escherichia coli* bacteria in planktonic state. BAC is a cationic surfactant, belonging to the family of QACs (quaternary ammonium compounds), with antibacterial properties often used in water treatment [25]. BAC gathered the attention of the scientific community because of its physical–chemical characteristics [26] and its mechanisms of interaction with bacteria [27], later discussed in the Results and Discussion section of the present paper. Several studies regarding the immobilization of QAC compounds in nanostructured material were published [22], including the functionalization of metal oxide nanoparticles [28,29]. However, none of those cases include the functionalization of macroscopic metal oxide pellets.

The present work is the first step to pursue the incorporation of antimicrobial macroscopic particles into a continuous flow bed-reactor (fixed or fluidized) to be added as an RO pretreatment step of a water stream. The success of this approach will contribute to optimize the application of biocides while minimizing the environmental and health impacts. Therefore, the solution here proposed considers the use of commercially available metal oxides (pellet shape, overall average dimension of 3 mm) with added biocidal properties, overcoming two issues brought by real-field applications of nanoparticles: the difficulty in removing nanoparticles from industrial-scale water streams and the high pressure drop caused by the compaction of a bed of nanoparticles.

## 2. Results and Discussion

### 2.1. Functionalization Characteristics

In order to activate the surface of alumina samples, the commercial materials were functionalized with a nitrogen precursor, namely dopamine. The nitrogen groups introduced in the particles’ surface act as anchorage points to immobilize the biocide. The prepared materials were characterized using different techniques, yielding the results described in the next subtopics.

#### 2.1.1. Thermal Stability of Functionalized Metal Oxides

**Figure 1:**
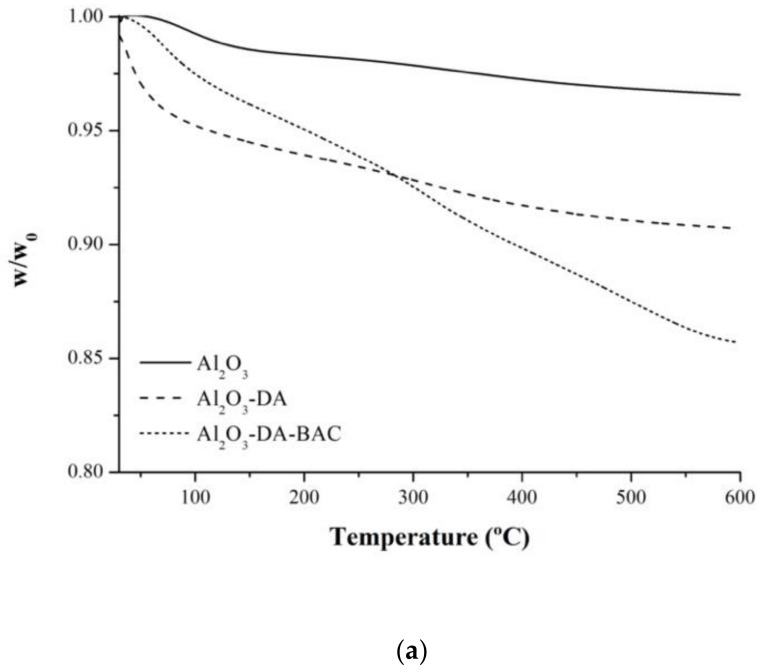

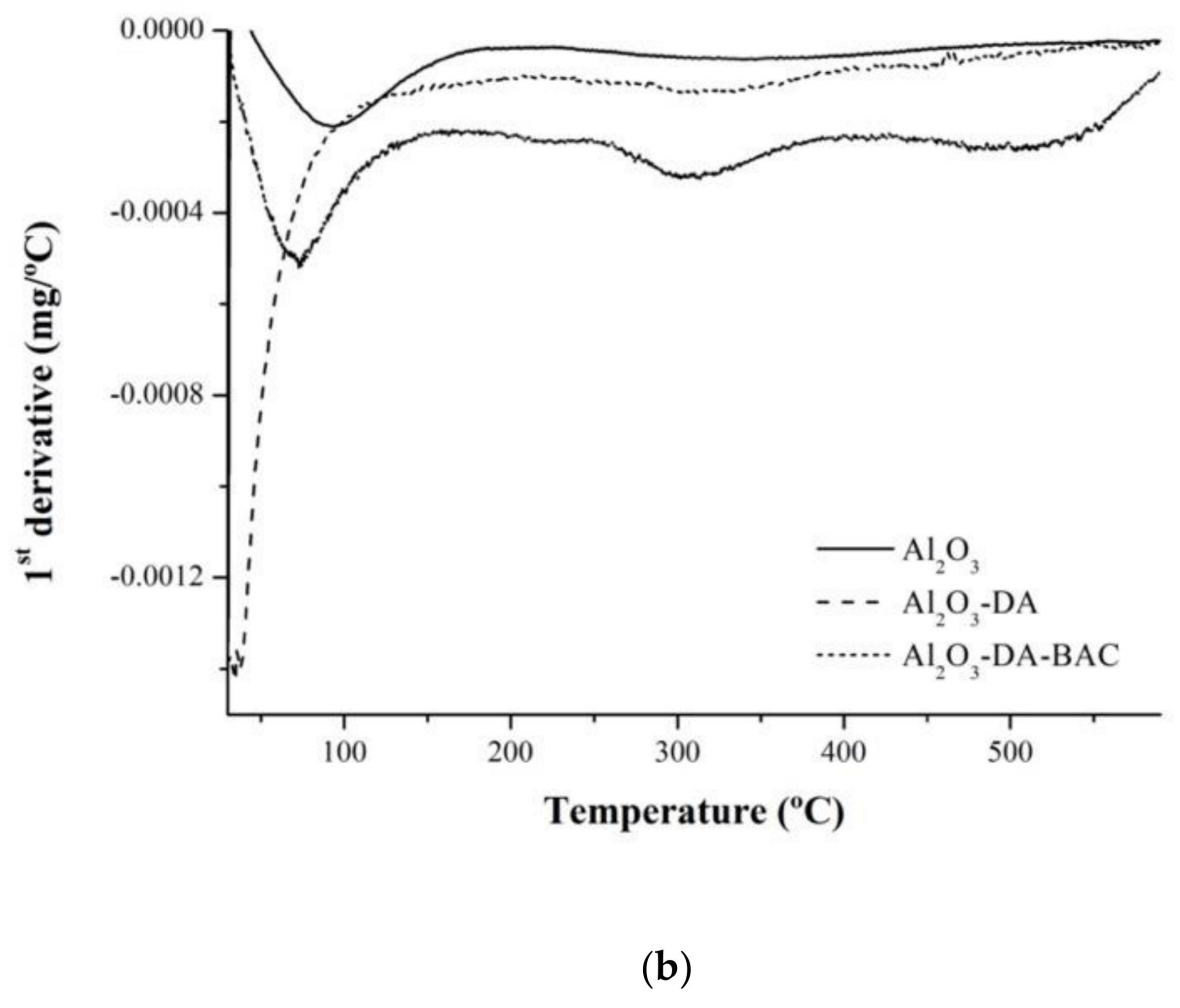
Thermogravimetric analysis, TG (**a**) and first derivative curve, DTG (**b**) of functionalized and Al_2_O_3_ particles.

To get more insight into the packing density, thermogravimetric analysis was used to estimate the amount of functional groups anchored to the surface of the particles (Figure 1). By observing the weight loss profiles of the raw material (Al_2_O_3_), it appears that the latter does not have organic impurities, because the values of burn-off obtained for these samples are very low (3%), see Figure 1a.

The functionalization with nitrogen precursors increased the values of the burn-off. The sample Al_2_O_3_-DA present weight losses between 200 and 400 °C of around 2%. The thermogravimetric analysis (Figure 1a) confirmed the success of the functionalization with nitrogen precursors. The interaction between dopamine and inorganic materials is most likely due to hydrogen bonding, conferring a high thermal stability to the sample.

The nitrogen groups introduced in alumina particles by dopamine will be the anchor points for BAC immobilization. It can be seen in Figure 1b that the Al_2_O_3_-DA-BAC sample presents a total mass loss of 3% in the temperature ranges of 280–390 °C and 426–598 °C. This 3% corresponds to the amount of immobilized BAC per unit mass of particles, as described in Section 3.4.3.

#### 2.1.2. Adsorption/Desorption Isotherms

**Figure 2:**
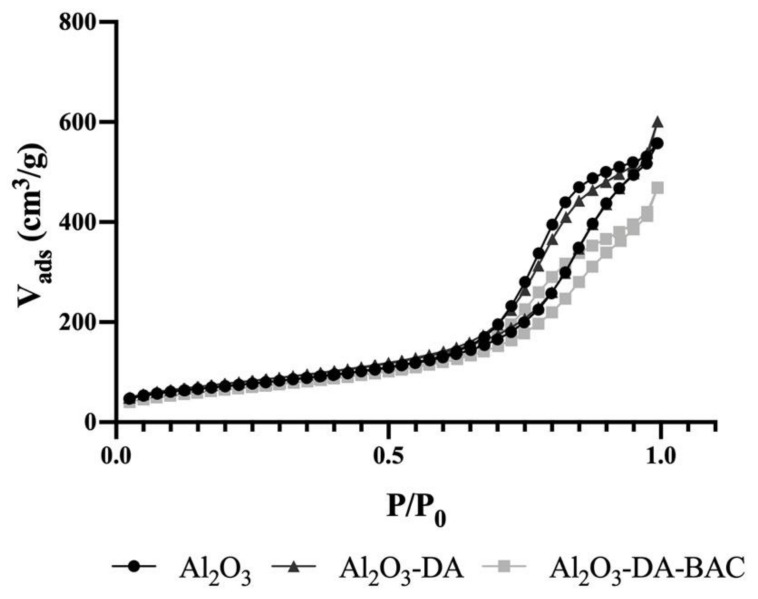
Nitrogen adsorption and desorption isotherms of functionalized and Al_2_O_3_ particles.

Adsorption and desorption isotherms of the particles were performed. As can be seen in Figure 2, all particles showed a type IV isotherm behavior, which indicates the presence of mesopores [30,31,32].

Table 1 summarizes the results obtained from nitrogen adsorption–desorption isotherms (Figure 2). According to the N_2_ physisorption, it appears that the functionalization treatment changed the textural properties of alumina materials. The surface area of the sample treated with dopamine increases after the treatment (from 258 to 280 m^2^/g). The development of porosity during the treatment with dopamine (Table 1), observed for the alumina materials, is due to the widening of existing pores (d_p_ increased from 8.2 to 9.0 nm), confirming the successful functionalization of this material. This can be explained by the fact that the dopamine solution has a high pH value (basic solution), which can cause some dissolution/etching of the aluminum oxide. After the BAC immobilization, the surface area of this sample decreased to 240 m^2^/g which can be explained by the occupation of some pores by the BAC molecules, since the pore volume decreased from 0.77 to 0.60 cm^3^/g.

#### 2.1.3. X-ray Diffraction (XRD) Analysis

**Figure 3:**
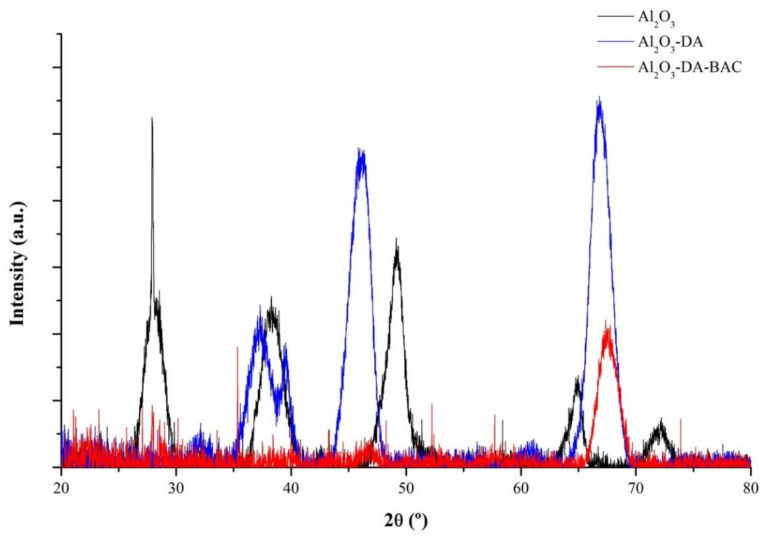
X-ray diffraction (XRD) pattern of functionalized and Al_2_O_3_ particles.

The XRD pattern of the alumina particles is represented in Figure 3. The original alumina patterns show relatively strong peaks at 2θ values at 28, 38, 49, 65 and 72°, which are attributed to the reflections of Boehmite (γ-AlO(OH)). The DA-functionalized sample presents a crystalline phase of ƞ- Al_2_O_3_, detected by the presence of peaks at 37, 39, 46 and 67°. Additionally, Al_2_O_3_-DA-BAC particles show an entire transformation to γ-Al_2_O_3_, detected by the presence of two peaks at 47 and 68°. Data from the XRD also confirms the successful functionalization of the particles with biocide.

#### 2.1.4. Point of Zero Charge—pH_PZC_

**Figure 4:**
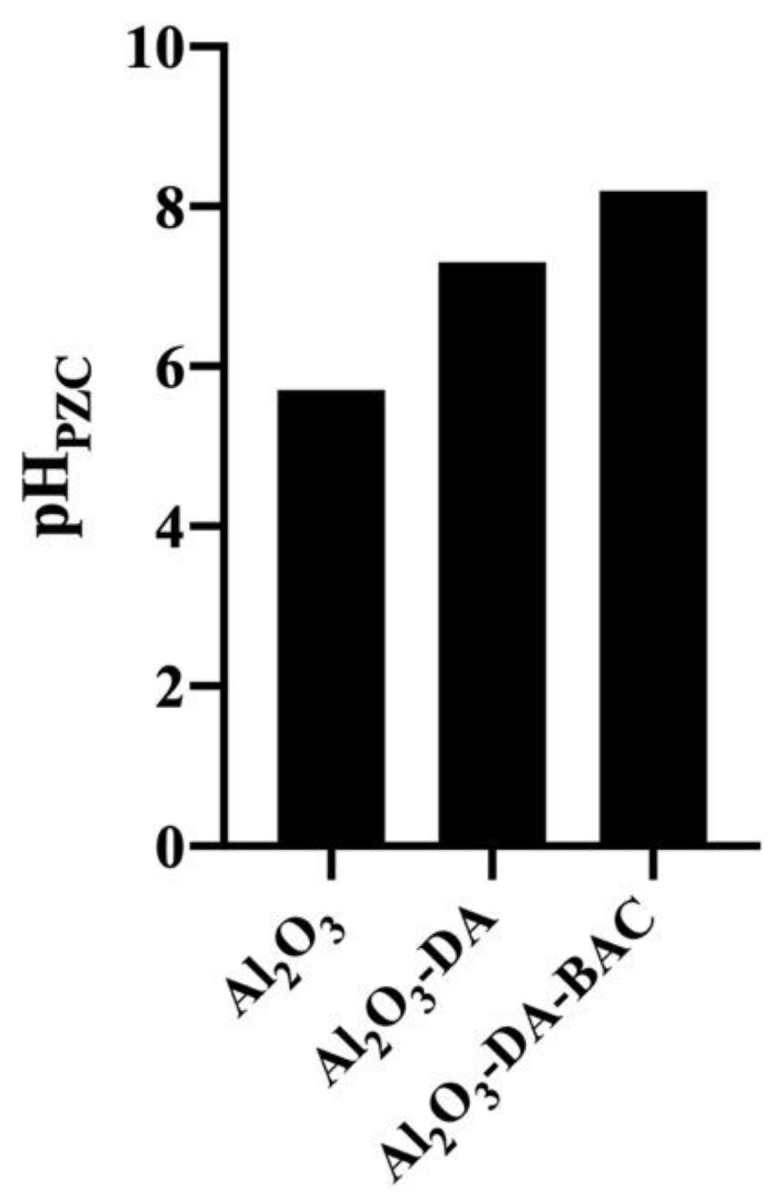
Point of zero charge of the functionalized and Al_2_O_3_ particles.

Figure 4 shows the pH_PZC_ values estimated for the alumina samples (prior to functionalization), which is found to be 5.7. This value increases to 7.3 after introduction of –NH_2_ groups, suggesting that the metal oxide surfaces have been modified with positively charged amino groups.

Furthermore, after BAC incorporation, the pH_PZC_ raised to 8.2. The basic nature of benzalkonium chloride corroborates the pH_PZC_ increase.

#### 2.1.5. Fourier-Transform Infrared Spectroscopy (FTIR) Analysis

**Figure 5:**
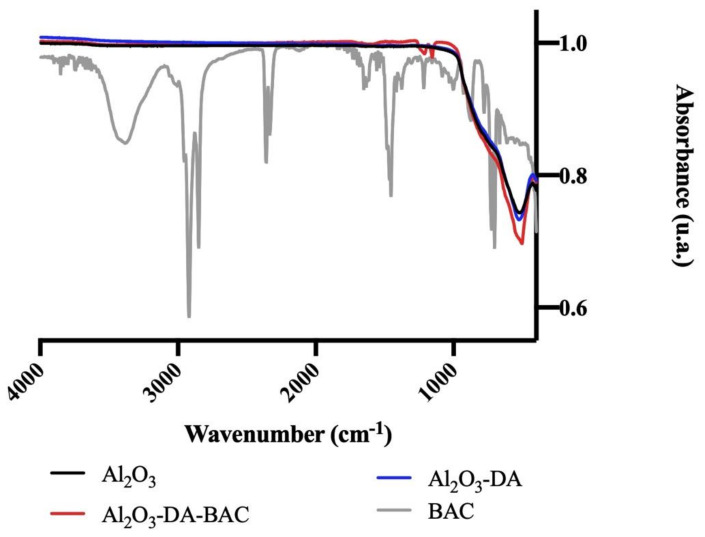
Fourier-Transform Infrared Spectroscopy (FTIR) spectra of functionalized and Al_2_O_3_ particles. Benzalkonium chloride (BAC) in solution was used as control.

FTIR spectra of Al_2_O_3_ particles (Figure 5) show a band in the region of 3300–3700 cm^−1^, which corresponds to the water O-H stretch [33]. Ribena [34] also found bands in the region 3000–3400 cm^−1^ after coating the surface of its particles with dopamine and attributed it to the intermolecular hydrogen bonds (O-H, N-H and aromatic CH2 stretching vibrations) naturally occurring between dopamine molecules. Regarding Figure 5, another band was found in the region of 1580–1650 cm^−1^ for Al_2_O_3_-DA and Al_2_O_3_-DA-BAC particles. This band corresponds to N-H bending [33], meaning that functionalization with dopamine coated the particles with amine groups. The functionalization of particles with BAC resulted in the appearance of two small peaks at 1153 and 1211 cm^−1^ on the curve in red. These peaks are associated to C-N and N-CH_3_ stretching vibrations, which are characteristic of quaternary amines [35,36].

FTIR results proved again the successful functionalization of particles with nitrogen and biocidal groups.

The overall physical and chemical characterizations discussed above (BAC peaks in the FTIR diagram, mass loss in the TG curves, XRD analysis and the sorption isotherms) point-out to a successful immobilization of active BAC groups after the proposed functionalization.

### 2.2. Particles’ Antimicrobial Activity

To assess the *Escherichia coli* susceptibility to the immobilized biocide, zones of inhibition were determined. Data shown in Table 2 demonstrate that Al_2_O_3_ and Al_2_O_3_-DA particles do not induce any zone of inhibition, suggesting that the core materials without biocide, for the present test conditions, have a very small or no antibacterial effect on *E. coli*. On the other hand, Al_2_O_3_-DA-BAC particles exhibited a zone of inhibition diameter of 18 ± 2 mm, which implies that there is some biocide leaching from the particles. Hassan and Elbagoury [37], while studying the susceptibility of some bacterial isolates to BAC in solution, observed similar inhibition diameters. These authors found zone of inhibition average diameters of 13 and 17 mm when *Pseudomonas* spp. cells were exposed to a 2% BAC solution.

The formation of a zone of inhibition is related to the diffusion constant of the antimicrobial agent [38,39,40] and, therefore, can be related to its amount and motility. According to the same authors [38,39,40], a zone of inhibition appears when the concentration of an antimicrobial agent exceeds the minimum inhibitory concentration or its critical concentration [38,39,40,41]. Although, this test highly depends on several experimental factors (e.g., test medium, organism and its microbial concentration and biocide type), it is expected that for the same experimental conditions, zones of inhibition can provide a qualitative indication of the bacterial susceptibility against a given antimicrobial agent [42]. Therefore, the results in Table 2 suggest that the functionalized particles (Al_2_O_3_-DA-BAC) have a strong antibacterial activity potential.

### 2.3. Effect of Immobilized Biocide Concentration on the Particles’ Antimicrobial Activity

According to what was described in the Materials and Methods section, different ratios of particles per unit volume of solution, summarized in Table 3, were tested. It is important to note that the concentration of immobilized biocide per unit mass of each particle was 3% (*w*/*w*) of BAC, according to the data obtained in the thermogravimetric analysis (as described in Section 2.1.1). Therefore, the tested concentrations of 500, 1000 and 3000 mg/L refer to the “overall concentration of immobilized biocide” defined as mg of immobilized biocide per L of total liquid volume (bacterial suspension). Thus, the different concentrations correspond to different amounts of particles per unit liquid volume.

The antimicrobial effect of such functionalized particles (Al_2_O_3_-DA-BAC), considering the different overall concentrations of immobilized biocide, was evaluated according to the bacteria culturability and membrane integrity (propidium iodide, PI, uptake percentage) criteria.

#### 2.3.1. Culturability

**Figure 6:**
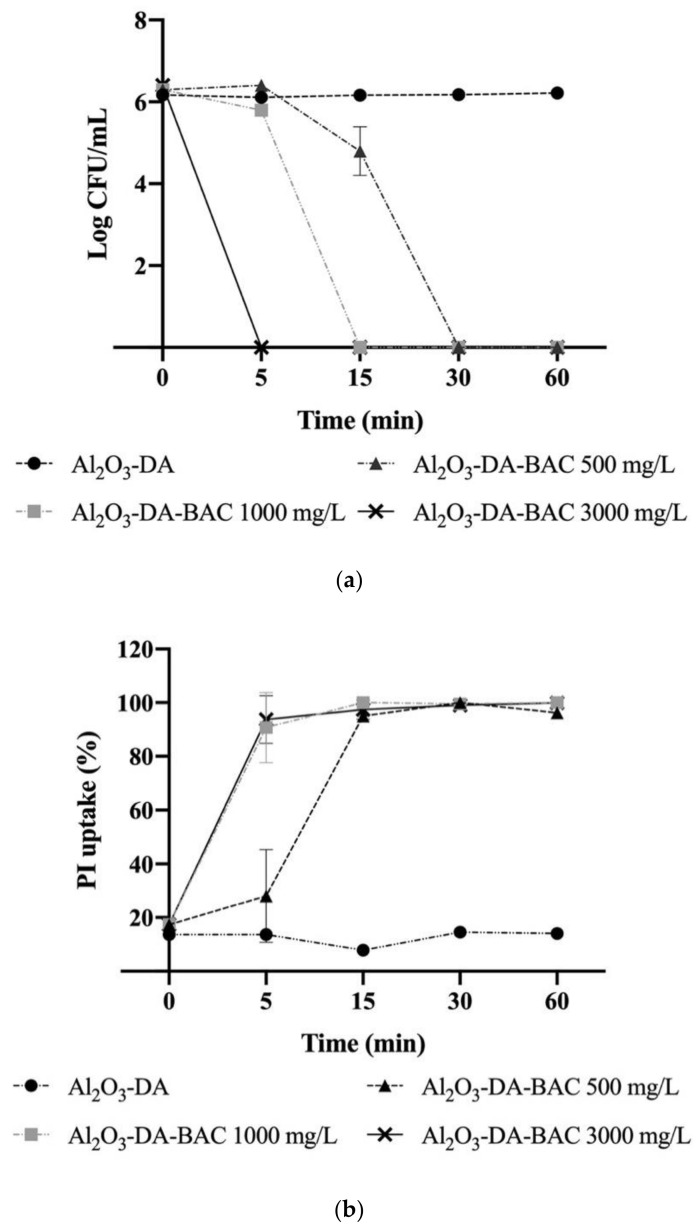
Logarithm of the colony forming units per milliliter (CFU/mL) (**a**) and propidium iodide (PI) uptake (%) (**b**) of *Escherichia coli* planktonic cells exposed to dopamine (DA)-functionalized particles over time during the first use. The overall tested concentrations were: 500, 1000 and 3000 mg/L. of Al_2_O_3,_ and Al_2_O_3_-DA particles were used as controls. Error bars correspond to the standard deviation of the mean determined for two independent experiments.

The reduction of the colony forming units per milliliter (CFU/mL) of *E. coli* planktonic cells over time, exposed to different overall concentrations of Al_2_O_3_-DA-BAC particles, is shown in Figure 6a. In this figure, it can be seen that higher concentrations of overall immobilized biocide require less contact time to completely inactivate bacterial cells. For instance, a statistically significant (*p* < 0.05) six-log reduction (until no CFU count) was observed after a five-min contact time when using the overall biocide concentration of 3000 mg/L. The contact time required to obtain no CFU count was respectively 15 min and 30 min for 1000 mg/L and 500 mg/L immobilized biocide concentrations.

Furthermore, Figure 6a shows that the control particles (Al_2_O_3_-DA) had no impact on the culturability, since no decrease on CFU/mL was observed. Statistically significant differences (*p* < 0.05) were found between the biocidal particles (at all the tested concentrations) and the control particles, confirming the effective bactericidal activity of the BAC-loaded particles and the non-existing antimicrobial activity of the core–base materials. Although some studies report the antimicrobial activity of dopamine, such antimicrobial activity seemed to be related with the high concentration of DA tested. For example, Zhao et al. [43] reported that at 100 mg/mL of DA coating had an antimicrobial effect against *E. coli* cells, while at 30 times lower concentrations, Iqbal et al. [44] found that DA did not exhibit any antimicrobial activity against the same bacterium. Indeed, the concentration in the Al_2_O_3_-DA particles in this study is only 2 mg/mL of DA, which agrees with the idea that low concentrations of DA do not have an antimicrobial effect.

#### 2.3.2. Membrane Integrity

The effect of the functionalized particles over time on the membrane integrity (represented in terms of PI uptake %) of the bacterial cells was also studied for different biocide concentrations- see Figure 6b. As expected, the impact of the control particles (Al_2_O_3_-DA, before functionalization), on membrane integrity loss was negligible when compared to the particles containing the immobilized biocide (*p* < 0.05).

The results show that total loss of membrane integrity (100% PI uptake) was achieved with functionalized Al_2_O_3_-DA-BAC particles for all tested concentrations, although the lower biocide concentration (500 mg/L) took some more time to reach very high levels (above 90%) of cell membrane permeabilization. It could be said that increasing the overall immobilized biocide concentration decreases the contact time needed to fully permeabilize bacterial membrane and reduce culturability. This is in accordance to what is widely described in the literature concerning the concentration and contact time of free biocides [45,46]. Additionally, Pazos-Ortiz et al. [47] found that bacterial inhibition was dose-dependent, when studying the antimicrobial activity of silver nanoparticles. However, the results obtained in the present work, concerning a liquid biocide incorporated in inert particles (similar curves for 1000 and 3000 mg/L, with faster effects than for the lower concentration of 500 mg/L) leave some doubt about the possible existence of a critical concentration above which the biocide concentrations remain equally efficient.

The conclusions are also not so straightforward when comparing culturability and membrane permeabilization. Looking at Figure 6a,b, a similar level of membrane permeabilization is observed at five min for concentrations of 3000 mg/L and 1000 mg/L, but the CFU log reduction is much more pronounced for the higher concentration than for 1000 mg/L. These discrepancies suggest that the membrane damage caused by the antimicrobial agent was not enough to affect the bacterial growth on solid media, depending on the biocide concentration.

There are several studies in the literature reporting differences between these two methods, mostly related to the existence of more viable than culturable cells. One of such examples is the work of Ferreira, et al. [48], who observed that some cells with intact membranes (no PI uptake) were not culturable—the so called “viable but nonculturable” state. Nonetheless, it has also been reported by Rosenberg et al. [49] that PI-based viability tests can overestimate the number of dead cells, since they found that a dual-species 24-h biofilm presented 96% of PI-positive cells, although 68% of those cells were still metabolically active, and more than 80% of these cells were cultivable after harvesting. Furthermore, it has been shown that membranes from cells exposed to stress conditions (starvation, heat, biocide, etc.), tend to become more permeable to PI, resulting in mistakenly marking viable cells as dead [50,51,52]. Interestingly, it was also found that if such cells were re-incubated for a certain period after being exposed to the stress conditions, they could recover and repair the membrane damage [50].

The results discussed herein suggest that membrane integrity assays do not correlate with the ability of bacteria to grow on solid medium. Therefore, they confirm the importance of complementing the “viability” assays methods with a cultivation method [53] or to incubate the cells prior to the membrane integrity staining procedure [50].

### 2.4. Effect of Particle Reuse on Their Antimicrobial Activity

**Figure 7:**
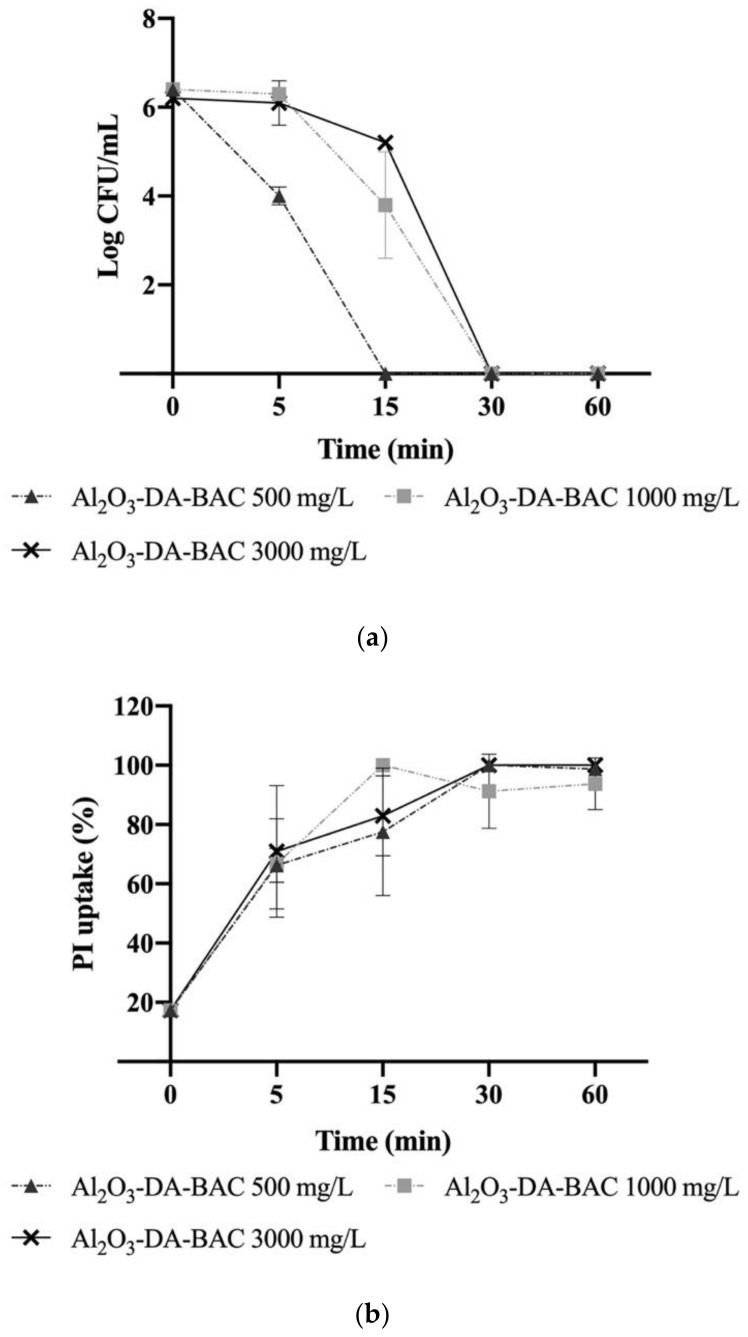
Logarithm of the colony forming units per milliliter (CFU/mL) (**a**) and propidium iodide (PI) uptake (%) (**b**) of *Escherichia coli* planktonic cells exposed to functionalized particles over time after reuse. The overall tested concentrations were: 500, 1000 and 3000 mg/L. Error bars correspond to the standard deviation of the mean determined for two independent experiments.

After being used once (first use), the same particles were reused and their antimicrobial activity regarding culturability (Figure 7a) and PI uptake (Figure 7b) was re-evaluated. In general, the particles lost some performance upon reuse, i.e., reusing the particles at 3000 and 1000 mg/L required more time to reach the maximum efficiency in terms of CFU count and % of PI uptake. This issue is addressed in Section 2.5, where the mechanisms of particles’ antimicrobial activity are discussed.

However, there is a specific case that stands out: Al_2_O_3_-DA-BAC at 500 mg/L. In this case, at first sight there would be an improvement of the particles’ performance on reuse, since the contact time until total inactivation seems to decrease from 30 min on the first use to 15 min upon reuse. This can be related to the large period between sampling time points. Most probably, the total inactivation might occur at min 16 or 17. This would not be detected in the present tests, but the actual performance of the Al_2_O_3_-DA-BAC at 500 mg/L would be the same in the first use and in reuse.

Similar to what was observed in the first use, the PI uptake percentages (Figure 7b) do not match with the CFU results (Figure 7a). For example, regarding Al_2_O_3_-DA-BAC at 500 mg/L, although the cells lost their culturability at 15 min, 22% of them did not present damaged membranes. Possibly, this may be related to the fact that the cells have reached the “viable but non-culturable” state [49].

**Figure 8:**
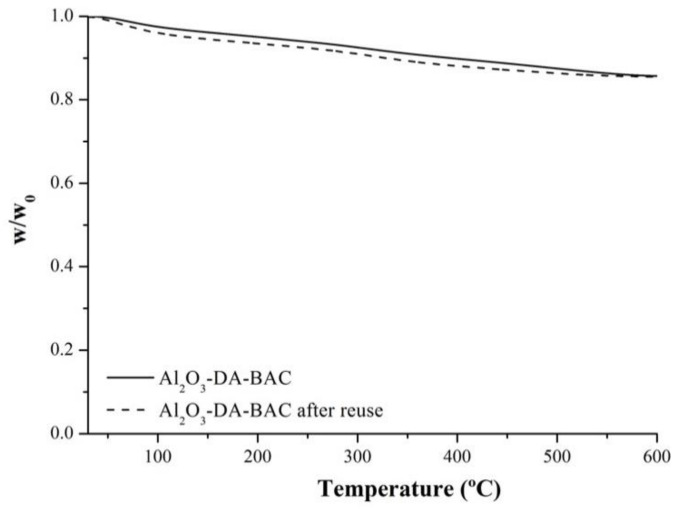
Thermogravimetric analysis (TG) of Al_2_O_3_-DA-BAC particles before the antimicrobial experiments and after being reused.

Due to the differences observed after reuse, a TG analysis of the particles after being reused was performed (see Figure 8). It is important to refer that the samples used were particles from the 3000-mg/L experiments.

From Figure 8, it is possible to see that the curves are very similar. The amount of BAC in the samples after use is still in the order of 3% (*w*/*w*), meaning that there was no significant release of BAC. The results from the zone of inhibition seem to indicate a higher release, but it is known that the biocide/antibiotic concentrations in agar are higher than in liquid medium, causing higher death rates [54]. The zone of inhibition is only a qualitative method, while TG gives quantitative results. Furthermore, in the inhibition zone experiments the particles are in contact with bacteria for 24 h, which is much longer than the contact time of the killing experiments (one hour). Gathering all data, it is possible to conclude that there is a small and slow release of biocide from the particles.

### 2.5. Hypothesis for the Underlying Mechanism of Action of Immobilized Biocide

The results observed after reuse can be related to the following main question: “what are the mechanisms of antimicrobial action of biocide-loaded particles?” There are some hypotheses, namely: (i) bacteria are inactivated by the free biocide resulting from the slow release to the bulk solution, (ii) direct contact with the immobilized biocide (also known as “contact killing”) is responsible for the antimicrobial action and (iii) there is a combination of both mechanisms of action stated above.

If the first hypothesis was behind the antimicrobial action of the particles, the reuse should have a similar behavior. Since the amount of biocide immobilized per unit mass of particle is very high, it would be expected that upon reuse there would still remain a considerable amount of biocide to be released. Additionally, the hypothesis that all or most of the immobilized biocide could be released during the first use is not plausible, otherwise the killing kinetics for the 3000 and 1000 mg/L concentrations would be faster.

The second hypothesis is that bacteria can be killed by contact with the BAC molecules immobilized onto the particles surface [54,55]. Several authors have been developing contact-active materials, which consist of coating a surface with a biocidal layer [56,57,58]. Synthetic QACs are by far the most used molecules for this purpose [59]. The mechanism of antimicrobial action of QACs seems to be due to electrostatic interactions between the positively charged QAC molecules and the negatively charged phospholipids present in the cytoplasmatic membrane of bacteria. Furthermore, the hydrophobic alkyl chain of QACs punches the membrane, leading to membrane disruption, cellular content leakage and microbial death [59,60,61]. Moreover, for the interaction between BAC molecules and bacteria to occur, the particles need to be in close contact with bacteria. Of course, the BAC molecules inside the pores will not be readily available for bacteria [62], since the particles are mesoporous (pore diameters between 2 and 50 nm [63]) and bacteria (due to their bigger size) cannot enter the pores. In the work of He et al. [56], they found that bacteria are dead upon contact with the immobilized QACs. However, it is known that these antimicrobial moieties can be blocked by dead adherent bacteria [56,64]. This mechanism of contact killing and consequent biocide blocking by dead bacteria may apply to the results observed in our study. Attraction between the particles, which display positive surface charge at pH around 7 (because the isoelectric point is pH = 8.2; see Figure 4), and the negatively charged bacterial surfaces is most probable to occur. It is therefore plausible that some dead bacteria from the 1^st^ use remained attached to the particles’ surface, which would explain the performance decrease upon reuse.

The third hypothesis assumes that the mechanism of action is a combination of the first and the second hypotheses. In fact, as it was already discussed, the first theory alone is not plausible, but the results from the zone of inhibition show some biocide leaching, which cannot be neglected and could contribute to some (although low) antimicrobial action of the biocide in solution.

Work is now being carried out in order to fully understand these interactions between functionalized particles and bacteria. The implementation of additional analytical methodologies to deeply characterize the mechanism of action of the immobilized BAC and compare it with the mechanism of free biocide seems also to be a key step for further understanding of the results. Finally, assuming that particles lose some of their performance, several cycles of reuse assays should be performed in order to characterize their lifetime effectiveness. If so, for real-field applications, a particle regeneration (cleaning) step would be periodically needed, implying the simultaneous operation of two or more alternating parallel particle bed systems.

## 3. Materials and Methods

### 3.1. Reagents

Dopamine (3-Hydroxytyramine Hydrochloride) was obtained from Tokyo Chemical Industry Co., Ltd. (Tokyo, Japan). Benzalkonium chloride ≥95 was obtained from Sigma-Aldrich (Steinheim, Germany). Tris base was obtained from ChemCruz™ Biochemicals (Dallas, Tx, USA).

### 3.2. Preparation and Functionalization of Particles

Alumina oxide pellets (Al_2_O_3_, Saint-Gobain, Stow, MA, USA) with approximately 3 mm were selected as start material. Pellets activation was accomplished in a heat treatment at 600 °C under air atmosphere for 6 h.

#### 3.2.1. Initial Functionalization of Alumina Oxide Particles with DA

In this work, an amino-functionalization strategy was tested using dopamine (DA) as intermediate for BAC immobilization.

Dopamine hydrochloride (2 mg/mL) was dissolved in Tris buffer solution (pH = 8.5, 50 mM). The particles (5 g) were placed in contact with 50 mL of dopamine solution and left in agitation for 24 h. These Al_2_O_3_-DA particles were washed with distilled water to neutral pH, dried for 24 h in the oven at 120 °C and stored in a desiccator.

#### 3.2.2. Immobilization of Biocide on the Surface of Alumina Oxide Particles

Samples of Al_2_O_3_-DA particles (2 g) were added to 100 mL of 5% (*w*/*v*) QAC solution and allowed to interact for 72 h at room temperature and with agitation speed of 140 rpm. The resulting particles (Al_2_O_3_-DA-BAC) were washed with distilled water to neutral pH, dried for 24 h in the oven at 80 °C and stored in a desiccator.

### 3.3. Particles Characterization

#### 3.3.1. Thermal Stability of the Particles

Thermogravimetric analyses (TG) were performed to determine the particles content in terms of functional groups, after each immobilization step. It has been performed on a Mettler-Toledo TGA/DSC 1 STAR system. The thermal stability of samples was evaluated by heating the different samples up to 600 °C at 3 °C/min under air atmosphere and monitoring their weight loss.

#### 3.3.2. Textural Characterization of the Particles

The textural characterization of the materials was based on the N_2_ physisorption adsorption–desorption isotherms, determined at −196 °C with Quantachrome Autosorb IQ2. The surface area (*S*_BET_) was calculated from nitrogen adsorption isotherms using the Brunauer, Emmet, and Teller (BET) equation [65]. Pore size distributions were obtained from the desorption branch of the isotherm using the Barrett, Joyner and Halenda (BJH) method [66].

#### 3.3.3. Structural Properties of the Particles

Phase composition of the samples was analyzed by means of powder X-ray diffraction using a PanAnalytical X Pert PRO diffractometer set at 45 kV and 40 mA, using Cu Kα radiation (*λ* = 1.541874 Å) and a PIXcel detector. Data were collected using Bragg-Brentano configuration in the 2*θ* range of 20 to 80 º with a scan speed of 0.01 °/s

#### 3.3.4. Determination of Particle Surface Charge

The Zeta potential measurements were carried out using Dynamic Light Scattering by a Nano Particle Analyzer SZ-100 (Horiba Scientific, Longjumeau cedex, France). The particles (in powder) were placed in ultrapure water and the pH was adjusted from 2 to 11 by using an appropriate amount of 0.1-M NaOH or 0.1-M HCl. After being sonicated for 30 min, the particles surface charge was determined. At least 5 measurements were performed for each sample and the measurements repeated in two different occasions.

#### 3.3.5. Fourier-Transform Infrared Spectroscopy (FTIR)

Spectroscopic measurements were performed with a Vertex 80v FTIR spectrometer (Bruker Optics) using Attenuated total reflectance (ATR) mode. The samples between 400 and 4000 cm^−1^ were analyzed with 64 scans averaging 4 cm^−1^ and two independent experiments were performed for each sample.

### 3.4. Particles Antimicrobial Activity

#### 3.4.1. Microorganism

Bacterial suspensions of *Escherichia coli* CECT 434 were obtained from an overnight growth at 37 °C and under agitation (120 rpm) in R2A broth medium with the following composition (per liter): 0.5 g glucose (CHEM-LAB, Zedelgem, Belgium), 0.5 g peptone (Oxoid, Hampshire, England, UK), 0.1 g MgSO_4_.7H_2_O (Merck, Darmstadt, Germany), 0.5 g casein hydrolysate (Oxoid, Hampshire, England, UK), 0.3 g sodium pyruvate (Fluka, Steinheim, Germany), 0.5 g starch (Sigma-Aldrich, Steinheim, Germany ), 0.5 g yeast extract (Merck, Darmstadt, Germany) and 0.4 g K_2_HO_4_P·3H_2_O (Applichem Panreac, Darmstadt, Germany).

#### 3.4.2. Antimicrobial Activity of the Particles against *E. coli*

The antimicrobial activity of the particles was tested against *E. coli* by using the Kirby–Bauer disk diffusion assay. The overnight culture was diluted to achieve 10^6^ CFU/mL. Then, 100 μL of the test suspension was swabbed on Plate Count Agar (PCA) plates. Afterwards, the particles were placed on the center of the inoculated agar plates. Antibacterial activity was assessed by measuring the diameter of the inhibition zone (mm)—a circular area around the particle where bacteria has not grown—on the surface of the plates. Two independent experiments with triplicates were performed for each case.

#### 3.4.3. Effect of Particle Functionalization and Biocide Concentration on Antimicrobial Activity

The antimicrobial activity of the biocidal particles was tested against *E. coli*. An overnight culture was centrifuged at 4000 rpm for 12 min. The pellets were resuspended in phosphate buffered saline (PBS) buffer solution, washed twice and resuspended in the same solution. The bacterial count was then adjusted to 10^6^ CFU/mL. The particles were incubated with the bacterial cells for 5, 15, 30 and 60 min. As stated in the Introduction section, the aim of this work is to gather critical information on the properties and performance of the biocidal particles to proceed with the design of an antimicrobial particle bed-reactor. Therefore, by varying the number of particles, three different ratios of mass of particles per volume of solution were tested, corresponding to the following overall immobilized biocide concentrations (mass of immobilized biocide per total volume of solution): 500 mg/L, 1000 mg/L and 3000 mg/L (Table 3). These ratios were established attending to the bed-reactor dimensions, the particles characteristics (dimensions and density) and considering the wall effect approach [12]. The overall tested concentrations also considered the volume of solution (which was kept the same in all assays: 25 mL) and the amount of immobilized BAC per unit mass of particles (3%). The amount of biocide incorporated onto the particles’ surface was determined by TG, by calculating the mass loss that occurred at the BAC decomposition temperature range.

The biocidal activity was evaluated in terms of culturability and membrane integrity due to propidium iodide (PI) uptake. After being exposed to the particles, the bacterial suspension was successively diluted in PBS and culturability was assessed on Plate Count Agar (PCA) plates using the drop plate method [67]. Thereafter, plates were incubated at 37 °C for 24 h and the colonies enumerated. The culturability results are expressed as logarithm of the Colony Forming Units per milliliter (CFU/mL), with bacterial detection limit being 10^2^ CFU/mL (2 log). For membrane integrity assessment, the LIVE/DEAD^®^ *Bac*light™ kit (Invitrogen) was used. This kit is composed by two nucleic acids stains: SYTO9™ and PI. The last compound only penetrates into cells with damaged membranes, staining them in red. SYTO9™ crosses all bacterial membranes, staining the cells with green color [48,68]. To implement the method, cells were diluted 1:10 in PBS and 1 mL aliquot was filtered on a 0.2-μm Nucleopore^®^ (Whatman) black polycarbonate membrane and stained with 250 μL of SYTO9™ and 250 μL of PI. The stain reagents reacted for 7 min in the dark at room temperature. After that, the excess reagents were filtered, and the membrane mounted on a slide with *Bac*Light mounting oil. The microscope, software, and the emission and excitation filters used were the same as described by Ferreira et al. [48]. The results were represented in terms of PI uptake percentage. Two independent experiments were performed for each case.

#### 3.4.4. Effect of Reuse on Particles’ Antimicrobial Activity

After the first use (1st use), the functionalized particles were washed with 1 L of distilled water and dried at 120 °C for 24 h. Then, the particles were reused once, and their antimicrobial activity was re-evaluated as described in Section 3.4.3. Hereafter, this second use of the particles will be named—reuse.

### 3.5. Statistical Analysis

Data obtained for culturability and membrane integrity were analyzed using the statistical software GraphPad Prism 8.0 (GraphPad Software, Inc., San Diego, California USA). The influence of biocide concentration and reuse were evaluated using a two-way ANOVA with Tukey’s multiple comparison test. Differences were considered relevant if *p* < 0.05. All the statistical analyses were performed using the GraphPad Prism 8 software (GraphPad Software, Suite, San Diego, California, USA).

## 4. Conclusions

Alumina oxide particles were successfully functionalized with the antimicrobial agent benzalkonium chloride (BAC). To the best of our knowledge, this was the first work that accomplished the immobilization of biocides on the surface of macroscopic metal oxide particles, using DA (dopamine) as a precursor for biocide fixation. The functionalized particles (Al_2_O_3_-DA-BAC) showed a consistent antimicrobial effect against *E. coli*, which increased with the overall immobilized biocide concentration. In general, there was some decrease in the particles’ performance on reuse, corresponding to an increase in the contact time required to reach similar results to the 1^st^ use (for the same conditions). Even so, a high antimicrobial activity was still achieved upon reuse, indicating the feasibility of the Al_2_O_3_-DA-BAC particles for water disinfection, as a final polishing step before Reverse Osmosis treatment. The total cost of the core particles and the functionalization methods here described is relatively low when compared to the cost of, for example, silver nanoparticles used in lab scale experiments and are expected to be substantially reduced when going to an industrial scale production. In order to optimize the performance of the functionalized particles, the mechanism of action of the immobilized biocide needs to be studied in detail to understand how the immobilized biocide interacts with bacteria, in comparison with the free biocide mechanisms. Additionally, the possible interactions between functionalized particles and dead bacteria (or dead cell components) should be further investigated and characterized.

## Figures and Tables

**Table 1:** Surface area parameters obtained for the functionalized particles and the control (Al_2_O_3_).

Sample	S_BET_ (m^2^/g)	d_p_ (nm)	V_p0:0.95_ (cm^3^/g)
Al_2_O_3_	258	8.2	0.77
Al_2_O_3_-DA	280	9.0	0.77
Al_2_O_3_-DA-BAC	240	8.1	0.60

S_BET_—surface area, d_p_—pore diameter and V_p0:0.95_—pore volume.

**Table 2:** Zone of inhibition diameter obtained for the tested particles against *Escherichia coli.*

	Zone of Inhibition (mm)
Al_2_O_3_	0 ± 0
Al_2_O_3_-DA	0 ± 0
Al_2_O_3_-DA-BAC	18 ± 2

Values are presented as the mean ± standard deviation of two independent experiments.

**Table 3:** Tested conditions regarding the overall immobilized biocide concentration.

Particles	Immobilized BAC *w*/*w* (%): Mass of BAC (g) per 100 g of Particles	Ratio Mass of Particles (g)/Volume of Solution (L)	Overall Concentration under Test (mg of Immobilized Biocide/L Solution)
Al_2_O_3-_DA-BAC	3	16.8	500
		33.5	1000
		100.3	3000

## Data Availability

Not applicable.
